# Highly Efficient Photocatalytic Hydrogen Evolution over Mo-Doped ZnIn_2_S_4_ with Sulfur Vacancies

**DOI:** 10.3390/nano12223980

**Published:** 2022-11-11

**Authors:** Wei Guan, Lin Zhang, Peng Wang, Ying Wang, Haoyu Wang, Xingchen Dong, Ming Meng, Lina Sui, Zhixing Gan, Lifeng Dong, Liyan Yu

**Affiliations:** 1 College of Materials Science and Engineering, Qingdao University of Science and Technology, Qingdao 266042, China; 2 School of Physics and Telecommunication Engineering, Zhoukou Normal University, Zhoukou 466001, China; 3 Center for Future Optoelectronic Functional Materials, School of Computer and Electronic Information/School of Artificial Intelligence, Nanjing Normal University, Nanjing 210023, China

**Keywords:** photocatalytic hydrogen production, element doping, atomic vacancies

## Abstract

The introduction of impure atoms or crystal defects is a promising strategy for enhancing the photocatalytic activity of semiconductors. However, the synergy of these two effects in 2D atomic layers remains unexplored. In this case, the preparation of molybdenum-doped thin ZnIn_2_S_4_-containing S vacancies (Mo-doped Sv-ZnIn_2_S_4_) is conducted using a one-pot solvothermal method. The coordination of Mo doping and S vacancies not only enhances visible light absorption and facilitates the separation of photogenerated carriers but also provides many active sites for photocatalytic reactions. Meanwhile, the Mo-S bonds play function as high-speed channels to rapidly transfer carriers to the active sites, which can directly promote hydrogen evolution. Consequently, Sv-ZnIn_2_S_4_ with an optimized amount of Mo doping exhibits a high hydrogen evolution rate of 5739 μmol g^−1^ h^−1^ with a corresponding apparent quantum yield (AQY) of 21.24% at 420 nm, which is approximately 5.4 times higher than the original ZnIn_2_S_4_. This work provides a new strategy for the development of highly efficient and sustainable 2D atomic photocatalysts for hydrogen evolution.

## 1. Introduction

With the acceleration of global population growth and social development, environmental problems and energy deprivation emerged as two major issues that need to be addressed. Therefore, the search for alternative energy sources is of great significance to the development of society. H_2_ is considered as an ideal alternative energy source due to its clean, renewable and transportable advantages [1,2,3,4]. However, the conventional hydrogen production process has high energy consumption, low efficiency and serious environmental pollution, which seriously restrict the development of hydrogen. In contrast, photocatalytic fracking is a cleaner and is a more sustainable way of converting sustained solar energy into chemical energy. Its large-scale application will help alleviate problems such as the greenhouse effect and environmental pollution [5]. However, the low photocatalytic efficiency still remains a great challenge for practical applications. Therefore, in-depth research on hydrogen production by the photocatalytic cracking of water is of great significance [6,7,8,9,10].

In recent years, 2D semiconductors, such as C_3_N_4_, TiO_2_, CdS and MoS_2_, attracted much attention [11,12,13]. The two-dimensional matter shows a unique confinement of electrons in ultra-thin layers, resulting in superior optical and electronic properties. Two-dimensional ultrathin materials with suitable band gap structure have shown great potential in achieving efficient photocatalysis due to their unique structure and electronic properties. [14]. ZnIn_2_S_4_ is a classical trimetallic sulfide semiconductor with a tunable band gap of 2.06 eV to 2.86 eV. Due to its unique crystal growth mechanism and interatomic interactions, it is easy to form 2D layered structures. Currently, controllable morphologies include nanotubes [15], nanoribbons [16] and nanoflowers, and they are assembled from 2D thin nanosheets [17,18,19]. In addition, ZnIn_2_S_4_ has the advantages of being low in toxicity and possesses good photostability. However, similarly to most 2D semiconductors, ZnIn_2_S_4_ still has some problems in practical applications, such as the rapid recombination of light-generated electron-hole pairs, short carrier lifetime and low light absorption capacity.

To improve the photocatalytic activity of ZnIn_2_S_4_, a series of modifications have been carried out, including element doping, defect engineering, cocatalyst loading, morphology tuning and heterostructure construction [20,21,22,23,24]. Among them, element doping and defect engineering are considered the most effective means to enhance photocatalytic activities [25,26,27,28,29,30,31]. On the one hand, the concentration and energy distribution of carriers near the conduction band edge can be adjusted by doping the semiconductor with ions to introduce donor/acceptor energy levels, thus improving the electronic transition behavior [32,33,34,35,36,37]. For example, Huang et al. reported Mo-doped ZnIn_2_S_4_ flower-like hollow microspheres for efficient photocatalytic hydrogen evolution, and the results showed that the hydrogen evolution activity of Mo-doped ZnIn_2_S_4_ was 9 times higher (4.62 mmol g^−1^ h^−1^) than the pristine ZnIn_2_S_4_ (0.465 mmol g^−1^ h^−1^) [38]. They found that the doping of Mo into the ZnIn_2_S_4_ crystal lattices can introduce a doping energy level within the band gap. Therefore, the electronic conversion from the valence band to doping energy levels or from doping energies to the conduction band can effectively expand the light absorption range and improve the electronic conversion efficiency of solar energy. In addition, Mo doping can form Mo-S bonds, which accelerate the photogenerated carrier transport and separation, thus facilitating the transfer of light-excited carriers to the adsorbed molecules of reactants for photocatalytic reactions. A similar improvement in photocatalytic activity was also observed in Ni-doped ZnIn_2_S_4_ [39]. On the other hand, surface defects can lead to trapped photogenerated carriers, tuning their spatial distribution and prolonging their lifetime. Previous reports validated that S vacancies (Sv) enhances the absorption of visible light and adds to the photogenerated electric charge density, thereby enhancing photocatalytic activities [40]. For example, Yang et al. reported an Sv-containing half-cell ZnIn_2_S_4_, which exhibited a 7.8 times enhancement of photocatalytic hydrogen generation performance under exposure to visible light compared to pristine ZIS. Similarly, improved photocatalytic activities are also observed in ZnIn_2_S_4_ with In vacancies [41] and Zn vacancies [42]. Although metal doping and atomic vacancies have been employed separately to improve the photocatalytic action of ZnIn_2_S_4_, the coordination of these two effects is still unexplored.

Herein, a simple one-pot soluble heat method was developed to dope Mo into ZnIn_2_S_4_ nanoflakes containing Sv. An excess of thioacetamide (TAA) was added to the reaction. The adsorption of thioacetamide on the surface of the crystal hindered the growth of the crystal, leading to the formation of vacancy structure [43,44,45]. Meanwhile, Na_2_MoO_4_ was added to incorporate Mo atoms into the lattice of ZnIn_2_S_4_. The coordination of Mo doping and sulfur vacancies not only enhances the light absorption and the separation of photogenerated carriers but also provides a large number of active sites for photocatalytic reactions [46,47]. Therefore, the as-prepared Mo-doped Sv-ZnIn_2_S_4_ exhibited improved photocatalytic performance and stability. The photocatalytic hydrogen evolution rate of Sv-ZnIn_2_S_4_ with optimal Mo doping amounts reached 5.74 mmol g^−1^ h^−1^ under visible light irradiation, which is 5.4 times that of pure ZnIn_2_S_4_ and 2.6 times that of Sv-ZnIn_2_S_4_.

## 2. Results

**Figure 1:**
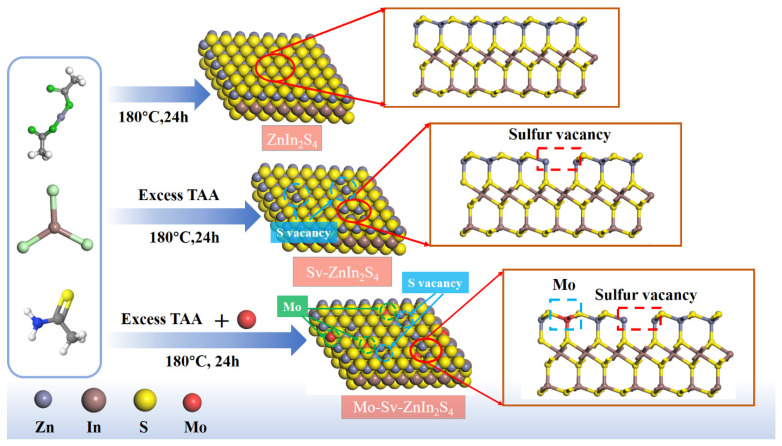
Schematic diagram of the synthetic routes for ZIS, Sv-ZIS and Mo-Sv-ZIS. The dotted red squares highlight the Sv. (The squares show the differences between the crystals).

**Figure 2:**
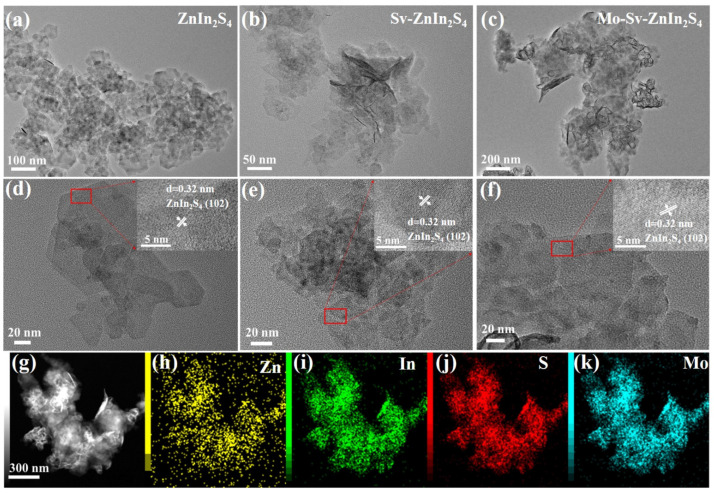
(**a**,**d**) TEM (**a**) and HRTEM (**d**) images of the ZIS. (**b**,**e**) TEM (**b**) and HRTEM (**e**) images of the Sv-ZIS. (**c**,**f**) TEM (**c**) and HRTEM (**f**) images of the 1.5% Mo-Sv-ZIS. (**g**) HAADF-STEM image of the 1.5% Mo-Sv-ZIS. (**h**–**k**) EDS element mappings of Zn (**h**), In (**i**), S (**j**) and Mo (**k**) in 1.5% Mo-Sv-ZIS. (Note: The area inside the red square is the area magnified by HRTEM).

As shown in Figure 1, ZIS, Sv-ZIS and Mo-Sv-ZIS were obtained by a simple single-pot soluble heat method. The number of sulfur vacancies was controlled by dosage of TAA. The amount of TAA during the synthesis was used to inhibit the growth of ZIS primary crystals, thus introducing Sv in ZIS [6]. The surface morphologies of the obtained ZIS, Sv-ZIS and Mo-Sv-ZIS are described by a field emission transmission electron microscopy (FE-TEM). As shown in Figure 2a,d, the basic morphology of the pristine ZIS is made up of a great number of two-dimensional hexagonal nanoflakes, which facilitates the acquisition of a large contact area to expose more reactive sites for the photocatalytic interaction. As shown in Figure 2b, the introduction of sulfur vacancies hardly changes the morphology. Sv-ZISs are also irregular flakes. The HRTEM image in Figure 2e shows that the crystal spacing between the planes of Sv-ZIS is 0.32 nm, which belongs to the (102) plane of ZnIn_2_S_4_. As shown in Figure 2c, after Mo doping, Mo-Sv-ZIS still maintains thin nanosheet structures. Due to the thinness of the nanosheets, there are different degrees of curling and bending (Figure S1). The HRTEM image shown in Figure 2f suggests that the interplanar crystal spacing of Mo-Sv-ZIS is 0.32 nm, which is similar to that of Sv-ZIS, indicating minor lattice distortions induced by Mo doping. The elemental distribution in the Mo-Sv-ZIS prototype is further studied by high-angle annular dark-field scanning transmission electron microscopy (HAADF-STEM) (Figure 2g). Figure 2h–k show the distribution of the corresponding elements (Zn, In, S and Mo) for Mo-SV-ZIS, and Figure S2 shows the corresponding energy dispersive spectra (EDS), showing the existence of Zn, In, S and Mo components. The uniform distribution of Mo indicates that it is doped successfully into the lattice of ZIS.

**Figure 3:**
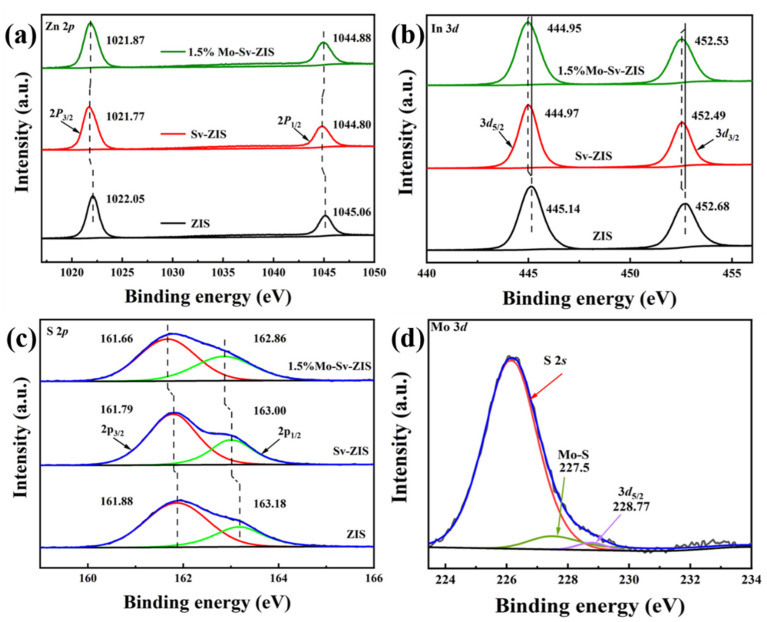
(**a**–**d**) High-resolution XPS spectra of Zn 2*p* (**a**), In 3*d* (**b**), S 2*p* (**c**) and Mo 3*d* (**d**) of ZIS, Sv-ZIS and 1.5% Mo-Sv-ZIS.

ZIS, Sv-ZIS and Mo-Sv-ZIS are investigated further by XPS for chemical states and apparent chemical compositions. Figure S3 displays the full spectrum of ZIS, Sv-ZIS and Mo-Sv-ZIS containing the typical peaks of Zn 2*p*, In 3*d*, S 2*p* and Mo 3*d*, which is consistent with the EDS’ results. Figure 3a shows that the Zn 2*p* characteristic peaks of ZIS are located at 1022.05 eV and 1045.06 eV, which belong to 2*p*_3/2_ and 2*p*_1/2_, respectively. In comparison, the Zn 2*p* XPS peak positions of Sv-ZIS undergo negative shifts of 0.28 eV and 0.26 eV, separately indicating a decrease in the coordination number of Zn atoms due to the presence of sulfur vacancies [5]. The doping of Mo shifted the peak positions of Mo-Sv-ZIS positively by 0.1 eV and 0.08 eV, which means that the Zn atoms returned to the higher binding energy region after the doping of Mo. As shown in Figure 3b, the In 3*d* characteristic peaks of ZIS are located at 445.14 eV and 452.68 eV, which are assigned to In 3*d*_5/2_ and 3*d*_3/2_, respectively. The In 3*d* peaks of Mo-Sv-ZIS and Sv-ZIS are essentially the same, with negative shifts of 0.19 eV and 0.15 eV, respectively. After the addition of Mo, the XPS peak position of In is almost unchanged, while the binding energy of Zn varies more than that of In, indicating that Mo replaces the position of Zn rather than that of In. The radius of Zn atom is 1.39 Å, and the radius of Mo atom is 1.40 Å, which are very close. Therefore, it is feasible to replace the Zn atom with the Mo atom. As shown in Figure 3c, the S 2*p* characteristic peaks of ZIS are located at 161.88 eV and 163.18 eV, which belong to 2*p*_3/2_ and 2*p*_1/2_, respectively. On the other hand, the S 2*p* XPS bands of Sv-ZIS underwent negative shifts of 0.09 eV and 0.18 eV, respectively, proving the existence of S vacancies in ZIS. After doping with Mo atoms, S 2*p*_3/2_ and S 2*p*_1/2_ negatively shift to 0.13 eV and 0.14 eV, respectively. The XPS results show that Mo doping leads to a further reduction in the coordination number of S in addition to the unpaired electrons brought by Mo. These two effects make the Mo-Sv-ZIS have a high density of unpaired electrons, which are active sites for photocatalytic reactions [48]. In addition, the atomic contents are calculated from the XPS peak areas. As shown in Table S1, the atomic ratios of Zn/In/S in ZIS, Sv-ZIS and Mo-Sv-ZIS are 1/1.6/3.5, 1/1.25/2.6 and 1/1.3/2.61, respectively. The lower S content in Sv-ZIS and Mo-Sv-ZIS supports the existence of a large number of S vacancies. In addition, the elemental content was tested by inductively coupled plasma mass spectrometry (ICP-MS) (Table S2). Moreover, the sub-band located at 227.5 eV due to the formation of Mo-S bonds is observed in the Mo 3*d* XPS spectrum [49], which further demonstrates the successful synthesis of Mo-doped Sv-ZIS (Figure 3d). The XPS O 1*s* peak of metal-O bonding typically is located at about 530 eV [50,51]. Herein, the O 1*s* peak positions of ZIS and Sv-ZIS are 532.36 eV and 532.17 eV, respectively (Figure S4), suggesting that there is no metal-O bonding.

**Figure 4:**
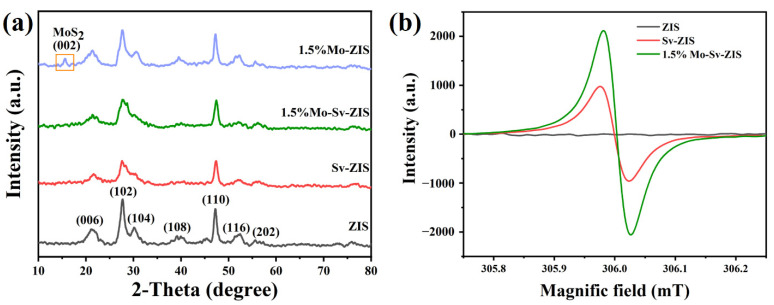
(**a**) XRD patterns of ZIS, Sv-ZIS, 1.5% Mo-ZIS and 1.5% Mo-Sv-ZIS. (**b**) The EPR Spectra of ZIS, Sv-ZIS and 1.5% Mo-Sv-ZIS.

The crystal structures of ZIS, Sv-ZIS and Mo-Sv-ZIS are investigated by powder X-ray diffraction (XRD). In Figure 4a, the XRD motif of ZIS is the same as that of ZnIn_2_S_4_ in hexagonal form (JCPDS file card No. 72-0773) without impurity peaks. The characteristic peaks at 21.6°, 27.6°, 30.5°, 39.8°, 47.1°, 52.2° and 55.6° belong to diffractions of (006), (102), (104), (108), (110), (116) and (202) crystal planes of ZnIn_2_S_4_, respectively. Among them, the diffraction peaks of (104), (108) and (116) crystal planes become weaker in the XRD of Sv-ZIS due to the presence of sulfur vacancies. The diffraction peaks of Mo-Sv-ZIS are similar to that of Sv-ZIS, which can prove the existence of sulfur vacancies in Mo-Sv-ZIS [39,41]. The XRD of Mo-ZIS is also measured for comparison. Interestingly, a diffraction peak at 15.5° is observed in the XRD of Mo-ZIS, which corresponds to the (002) planes of MoS_2_. Moreover, the diffraction of (104) planes at 30.4° is noticeable. When an appropriate amount of TAA is added, no additional S vacancies form in ZIS. The doped Mo atoms tend to bond with S atoms to form MoS_2_, which is not conducive to the full incorporation of Mo into the lattice of ZIS. In other words, the sulfur vacancies induced by the excess TAA can facilitate the doping of Mo into ZIS lattices. As illustrated in Figure S5, with an increase in Mo doping, the (006) and (110) crystal planes of Mo-Sv-ZIS shifted to high angles, demonstrating that the Mo atoms are included in the crystal lattice of ZIS [52,53]. The intensity of the individual diffraction peak diminishes with the increase in Mo doping. In addition, no significant peaks of diffraction of Mo species are found for the highly doped 5% Mo-Sv-ZIS. These results indicate that the Mo elements are uniformly incorporated into the lattice of ZIS and have not damaged the ZIS crystal structure. The number of unpaired electrons in ZIS, Sv-ZIS and Mo-Sv-ZIS are evaluated by electron paramagnetic resonance (EPR). As shown in Figure 4b, the original ZIS does not show any discernible signals. In contrast, Sv-ZIS shows an EPR indication for a g value of 1.998, confirming the existence of the S-vacancy. In addition, the EPR signal of Mo-Sv-ZIS is more intense, indicating that the increased number of unpaired electrons in Mo-doped Sv-ZIS is due to the simultaneous presence of undercoordinated Mo and S vacancies [54]. Therefore, the EPR results suggest that the density of active sites for photocatalytic reactions increased by Mo doping and S vacancies.

Furthermore, as shown in Figure S6, the analysis of the functional groups on the surface of the composites is performed by FTIR. The observed peaks at 1633 and 1394 cm^−1^ are composed of water and hypdroxyl moieties adsorbed on the surface [55], implying the ready attraction of free H_2_O molecules for continuous H_2_O dissociation and the facilitation of the reaction kinetics. Meanwhile, Raman spectroscopy is also performed to obtain more details about the crystalline structure. As shown in Figure S7, in the case of ZIS, the four Raman peaks are located around 253, 308, 339 and 370 cm^−1^, which are assigned to the longitudinal optical mode (LO_1_), transverse optical mode (TO_2_), longitudinal optical mode (LO_2_) and A1g mode of crystal ZIS, separately. For the Mo-Sv-ZIS, all peaks are relatively weak, and the peaks located at 308 and 339 cm^−1^ are barely observable because of the doping influence and the inferior degree of crystallinity. A new band at 405 cm^−1^, as shown in the Raman spectrum of Mo-Sv-ZIS, comes from the coupling of Mo-S stretching vibrations and A1g modes in Mo-Sv-ZIS, further confirming the existence of Mo-S bonds [5].

**Figure 5:**
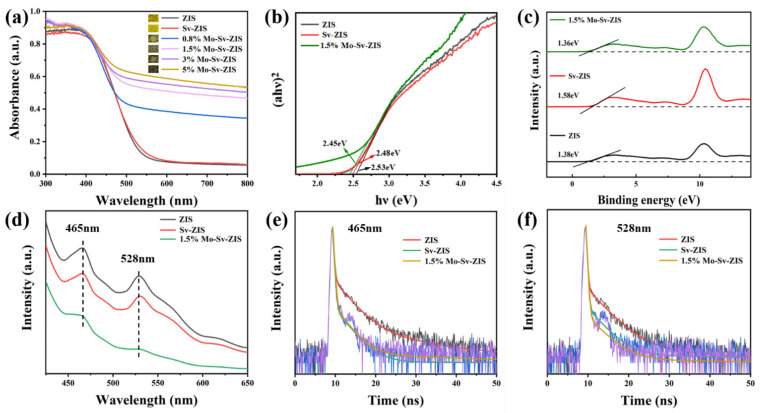
(**a**) UV–vis absorption spectra of ZIS, Sv-ZIS and x% Mo-Sv-ZIS (x = 0.8, 1.5, 3, 5). (**b**) (αhν)^2^ versus hν curves. (**c**) UPS spectra of the ZIS, Sv-ZIS and 1.5% Mo-Sv-ZIS. (**d**) PL spectra of ZIS, Sv-ZIS and 1.5% Mo-Sv-ZIS at 350 nm excitation. (**e**) Time-resolved PL spectra of ZIS, Sv-ZIS and 1.5% Mo-Sv-ZIS monitored at 465 nm. (**f**) Time-resolved PL spectra of ZIS, Sv-ZIS and 1.5% Mo-Sv-ZIS monitored at 528 nm.

The strong light absorption is beneficial to the generation of photogenerated carriers, which directly contribute to the improvement of photocatalytic performance. To investigate the optical absorption ability, UV-Vis spectra in the range of 300 to 800 nm are measured. As illustrated in Figure 5a, the primary light absorption of pristine ZIS and Sv-ZIS occurs in the wavelength range of 300–550 nm. With the introduction of S vacancies, the optical absorbance of Sv-ZIS is slightly enhanced compared to that of ZIS. After Mo doping, the optical absorption of Mo-Sv-ZIS further increased. With the increase in Mo content, the absorption in the wavelength range of 500 to 800 nm is significantly enhanced, and the color of the sample gradually deepens (inset in Figure 5a). Meanwhile, the absorption edge sightly redshifts. ZIS is a type of semiconductor with a direct band gap that can be computed from the following formula: ahv^2^ = A (hν- Eg). As shown in Figure 5b, the bandgaps of ZIS, Sv-ZIS and Mo-Sv-ZIS are 2.53 eV, 2.48 eV and 2.45 eV, respectively. Ultraviolet photoelectron spectra (UPS) are tested to determine the valence band position (E_VB_). As shown in Figure 5c, the valence band potentials (E_VB, XPS_) of ZIS, Sv-ZIS and Mo-Sv-ZIS are 1.38 eV, 1.58 eV and 1.36 eV, respectively. The E_VB_ of the corresponding standard hydrogen electrodes relative to ZIS, Sv-ZIS and Mo-Sv-ZIS are then obtained based on the following equation: E_VB, NHE_ = φ + E_VB, XPS_ − 4.44 eV; here, φ is the power functional of the apparatus (5.1 eV). Therefore, for ZIS, Sv-ZIS and Mo-Sv-ZIS, the E_VB, NHE_ are 2.04 eV, 2.24 eV and 2.02 eV, respectively [56].

The dynamics of excited carriers is studied through photoluminescence (PL) with time-resolved PL (TRPL). Two emission peaks at 465 nm and 528 nm are observed in the PL spectra of all samples, as indicated in Figure 5d. Among these three samples, ZIS has the highest PL intensity, which suggests an effective recombination of photogenerated carriers, greatly limiting the photocatalytic activity. However, the PL intensity of Sv-ZIS is obviously reduced, which suggests that the sulfur vacancy can act as a carrier trap and promote the separation of photocarriers [56]. It is clear that the PL intensity of the Mo-doped Sv-ZIS further decreases, indicating that photogenerated carriers can be also rapidly captured by the undercoordinated Mo, thereby significantly inhibiting the recombination of photogenerated electron-hole pairs. Time-resolved PL is tested at 465 nm and 528 nm (Figure 5e,f). The average PL lifetime (τ_A_) is calculated according to the following equation [48]:

$$\tau_{A}=\frac{A_{1}\tau_{1}{}^{2}+A_{2}\tau_{2}{}^{2}+A_{3}\tau_{3}{}^{2}}{A_{1}\tau_{1}+A_{2}\tau_{2}+A_{3}\tau_{3}}$$

where *τ*_1_, *τ*_2_ and *τ*_3_ are the PL lifetimes, and *A*_1_, *A*_2_ and *A*_3_ are the corresponding amplitudes obtained by tri-exponential fittings. The fitting details are listed in Table S3 and Table S4. The average PL lifetimes of the pristine ZIS are 5.33 ns and 3.31 ns for 465 nm emission and 528 nm emission, respectively. For the Mo-Sv-ZIS, the average PL lifetimes of 465 nm and 528 nm emissions shorten to 0.08 ns and 0.8 ns. The ultra-short PL lifetimes are in good agreement with the rapid capture of photogenerated carriers by the doped Mo and sulphur vacancies.

**Figure 6:**
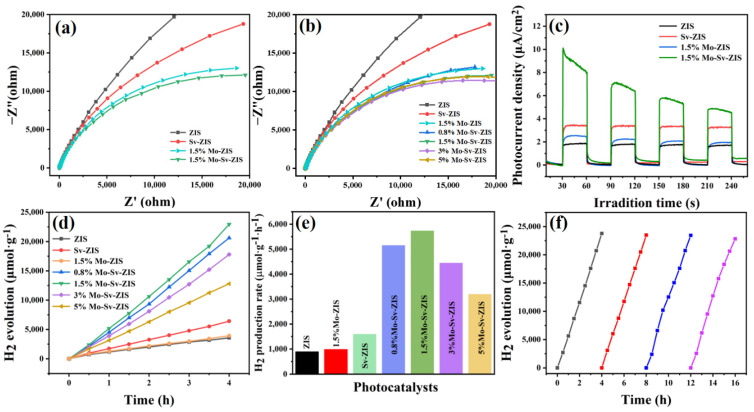
(**a**) The Nyquist plot displays the EIS of ZIS, Sv-ZIS, 1.5% Mo-ZIS and 1.5% Mo-Sv-ZIS. (**b**) EIS of all the samples. (**c**) Transient photocurrent responses of ZIS, Sv-ZIS, 1.5% Mo-ZIS and 1.5% Mo-Sv-ZIS electrodes under visible-light irradiation. (**d**) Time-dependent hydrogen evolution by photocatalysis in visible irradiation (>420 nm). (**e**) Photocatalytic H_2_ evolution rates of ZIS, Sv-ZIS, 1.5% Mo-ZIS and Mo-Sv-ZIS with different Mo contents. (**f**) Hydrogen evolution cycle 16 h test on 1.5% Mo-Sv-ZIS.

To investigate the role of Mo doping and S vacancies on photogenerated carrier migration, photoelectrochemical tests are carried out by using ZIS, Sv-ZIS, Mo-ZIS and Mo-Sv-ZIS as photoelectrodes. As shown in Figure 6a, the EIS radius of Sv-ZIS is smaller than the original ZIS, indicating that S vacancies can reduce the interfacial transfer resistance (detailed parameters are listed in Table S5). In addition, the EIS radius of Mo-Sv-ZIS is less than Sv-ZIS and ZIS due to the presence of both doped Mo and S vacancies [41,56]. The EIS spectra of Mo-Sv-ZIS with different Mo doping levels are further investigated. As shown in Figure 6b, the EIS semicircle becomes smaller as the Mo doping level increases. However, when the Mo doping concentration is as high as 5%, the radius of the EIS semicircle increases, implying that Mo doping that is too high is not conducive to lowering the surface charge’s transport resistance. As illustrated in Figure 6c, all tested samples exhibit evident photo-responses. As expected, Mo-Sv-ZIS exhibits the highest photocurrent density, which is four times higher than that of the pristine ZIS. Therefore, we demonstrate that the coordination of Mo doping and sulfur vacancies can significantly improve the photo-response of ZIS due to optical absorption enhancement, accelerated separation and the transport of carriers. The high photocatalytic activity of Mo-Sv-ZIS is expected in combination with the dense active sites.

The photocatalytic activities are assessed by hydrogen evolution in visible light (λ > 420 nm) irradiation. As shown in Figure 6d,e, the original ZIS shows poor hydrogen evolution activity of about 887 μmol g^−1^ h^−1^. Mo-ZIS and Sv-ZIS exhibit slightly higher hydrogen evolution activities of 983 μmol g^−1^ h^−1^ and 1607 μmol g^−1^ h^−1^, respectively. Among all the samples, the 1.5% Mo-Sv-ZIS shows the highest photocatalytic hydrogen evolution rate of 5739 μmolg^−1^ h^−1^, which is 5.4 time more than the original ZIS, 2.5 times better than that of Sv-ZIS, and 4.8 times higher than that of 1.5% Mo-ZIS. AQY value of 1.5% Mo-Sv-ZIS at 420 nm shows an excellent value of 21.24%. As summarized in Table S6, photocatalytic hydrogen evolution activities over 1.5% Mo-Sv-ZIS outcompete most reported photocatalysts based on ZnIn_2_S_4_. Moreover, Mo-Sv-ZIS has stable and sustainable photocatalytic activities. As shown in Figure 6f, the hydrogen evolution rate over 1.5% Mo-Sv-ZIS did not show a significant decrease after four periods. To verify the stability of the structure, XRD and XPS of the Mo-Sv-ZIS are tested after continuous photocatalytic hydrogen evolution for 16 h. As shown in Figures S8 and S9, both XRD and XPS results show negligible change after the long-term photocatalytic reaction, verifying the excellent stability of the Mo-Sv-ZIS.

**Figure 7:**
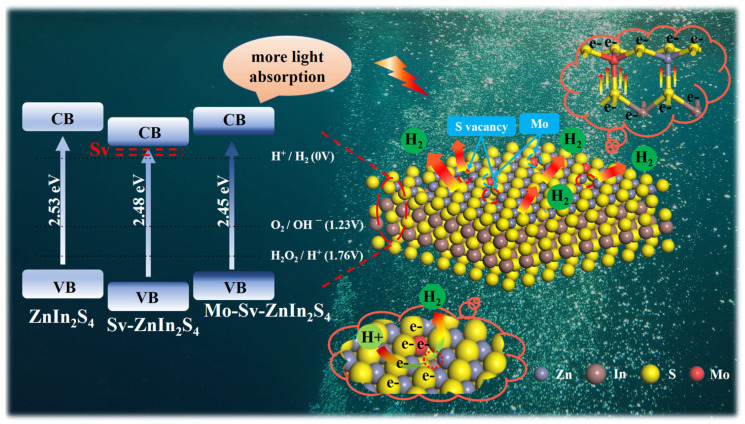
Schematic illustration explaining the excellent photocatalytic activity of the Mo-Sv-ZIS.

A scheme to explain the excellent photocatalytic activity of the Mo-Sv-ZIS is shown in Figure 7. The presence of S-vacancies and doped Mo atoms in ZnIn_2_S_4_ contribute to the change in electronic structure, which leads to a shift in the charged potential. In spite of the change in the position of both VB and CB, their CB potentials are still higher than the H^+^/H_2_ reduction potential and are still capable of photocatalytic hydrogen evolution. To gain more insight into the change of band positions, the density of states (DOS) of ZIS, Sv-ZIS and Mo-Sv-ZIS is calculated by using the Castep module of Materials Studio software (Figure S10). Under visible light irradiation, photogenerated electrons respond with H^+^ to form H_2_ ($H^{+}+e^{-}\to H_{2}$), while holes react with TEOA to form TEOA^+^ ($TEOA+h^{+}\to TEOA^{+}$). First, Mo doping significantly improves visible light absorption. Second, S vacancy and undercoordinated Mo can capture photogenerated carriers to inhibit electron-hole recombination. Third, the introduction of S vacancies and the doping of Mo atoms allow the ZIS system to have a lower coordination number, providing more reaction sites for photocatalytic reactions. Last but not the least, the formation of Mo-S bonds accelerates the charge transfer and reduce the internal resistance. As a consequence, Mo-Sv-ZIS exhibits an extremely high photocatalytic hydrogen evolution activity.

## 3. Conclusions

In summary, we develop a facile one-pot solvothermal method to synthesize Mo-doped 2D ZnIn_2_S_4_ nanoflake with S vacancies. Compared to pristine ZnIn_2_S_4_, the incorporation of Mo doping and S vacancies into the crystal structure modulates the electronic structure and photo-response of ZnIn_2_S_4_, resulting in a higher visible light absorption, faster carrier transfer rate and lower internal resistance. Meanwhile, the doping of Mo atoms with S vacancies reduces the coordination number of ZnIn_2_S_4_, gaining more active sites and, thus, accelerating the catalytic reaction. Thus, the hydrogen evolution rate of 1.5% Mo-Sv-ZIS reaches 5739 μmol g^−1^ h^−1^ under visible light irradiation, which is 5.4 and 2.5 times higher than that of pristine ZIS and Sv-ZIS, respectively. The corresponding AQY at 420 nm reaches 21.24%. Therefore, we demonstrate that the synergy between elemental doping and surface defects is an effective strategy to enhance the evolution of photocatalytic hydrogen in two-dimensional semiconductors, providing an additional perspective on the evolution of photocatalytic materials.

## Data Availability

Not applicable.

## Supplementary Materials

The following supporting information can be downloaded at: https://www.mdpi.com/article/10.3390/nano12223980/s1, Experimental section; Characterizations; Photoelectrochemical measurements; Photocatalytic hydrogen production; Calculation methods; Figure S1: (a–d) TEM images of 1.5% Mo-Sv-ZIS; Figure S2: EDS of the 1.5% Mo-Sv-ZIS shows the presence of Zn, In, S and Mo; Figure S3: Full survey XPS spectra of ZIS, Sv-ZIS and 1.5% Mo-Sv-ZIS; Figure S4: High-resolution XPS spectra of O 1s of ZIS (a) and Sv-ZIS (b); Figure S5: XRD patterns of ZIS, Sv-ZIS and 1.5%Mo-Sv-ZIS with different contents of Mo; Figure S6: FT-IR spectra of ZIS, Sv-ZIS and 1.5% Mo-Sv-ZIS; Figure S7: Raman spectra of ZIS and 1.5% Mo-Sv-ZIS; Figure S8: XRD patterns of the as- prepared 1.5% Mo-Sv-ZIS and 1.5% Mo-Sv-ZIS after photocatalytic test; Figure S9: XPS spectra of 1.5% Mo-Sv-ZIS after photocatalytic test. (a) Full survey XPS spectrum, (b) Zn 2*p*, (c) In 3*d*, (d) S 2*p* and (e) Mo 3*d*; Figure S10 The partial density of sates of (a) total orbit of ZIS and Sv-ZIS, (b) total orbit of Sv-ZIS and Mo-Sv-ZIS, (c) S atomic orbital of ZIS and Sv-ZIS, (d) Mo atomic orbital of Mo-Sv-ZIS; Table S1: Elemental compositions of ZIS Sv-ZIS and 1.5%Mo-Sv-ZIS according to XPS; Table S2 Element content of ZIS, Sv-ZIS and Mo-Sv-ZIS tested by ICP-MS; Table S3: Exponential decay-fitting parameters for time-resolved PL lifetime of ZIS, Sv-ZIS and 1.5% Mo-Sv-ZIS (465 nm); Table S4: Exponential decay-fitting parameters for time-resolved PL lifetime of ZIS, Sv-ZIS and 1.5% Mo-Sv-ZIS (528 nm); Table S5 Impedance values of ZIS, Sv-ZIS and 1.5%Mo-Sv-ZIS obtained by fitting on the EIS results; Table S6: Comparison of the 1.5% Mo-Sv-ZIS with other ZnIn_2_S_4_ related photocatalysts. References [38,39,40,57,58,59,60,61,62,63,64,65,66,67] are cited in the supplementary materials.
