# Supplementary material for: Highly Efficient Photocatalytic Hydrogen Evolution over Mo-Doped ZnIn2S4 with Sulfur Vacancies
Source: Nanomaterials (Basel). 2022 Nov 11;12(22):3980. doi: 10.3390/nano12223980 (PMC9695826; doi:10.3390/nano12223980)
Supplement: Supplementary file 1 [file nanomaterials-12-03980-s001.zip › nanomaterials-2001210-supplementary.pdf]

Supplementary Materials

Highly Efficient Photocatalytic Hydrogen Evolution over Mo-Doped $ZnIn_2S_4$ with Sulfur Vacancies

Wei Guan [1], Lin Zhang [1], Peng Wang [1], Ying Wang [1], Haoyu Wang [1], Xingchen Dong [1], Ming Meng [2], Lina Sui [1], Zhixing Gan [1, 3,*], Lifeng Dong [1,*] and Liyan Yu [1,*]

[1] College of Materials Science and Engineering, Qingdao University of Science and Technology, Qingdao 266042, China; qustgw@163.com (W.G.); buerne@163.com (L.Z.); w630502213@163.com (P.W.); qustwangying@163.com (Y.W.); wanghaoyu19990807@163.com (H.W.); 18563110769@163.com (X.D.); linasui@qust.edu.cn (L.S.)

[2] School of Physics and Telecommunication Engineering, Zhoukou Normal University, Zhoukou 466001, China; mengmingfly@163.com

[3] Center for Future Optoelectronic Functional Materials, School of Computer and Electronic Information/School of Artificial Intelligence, Nanjing Normal University, Nanjing 210023, China

* Correspondence: zxgan@njnu.edu.cn (Z.G.); donglifeng@qust.edu.cn (L.D.); liyanyu@qust.edu.cn (L.Y.)

Experimental section

Materials

$InCl_3$ (99.99%), $Zn(CH_3COO)_2 \cdot 2H_2O$ (99.8%), thioacetamide (TAA, 99%), triethanolamine (TEOA, 98%), Nafion solution (5wt%), Na_2MoO_4 (99%) were obtained from Shanghai Aladdin Ltd. Anhydrous ethanol (99.7%) were purchased from Sinopharm Chemical Reagent Co., Ltd. All chemicals were used as received without further purification.

Synthesis of ZIS and Sv-ZIS

The pure $ZnIn_2S_4$ (ZIS) nanosheets and $ZnIn_2S_4$ nanosheets with sulfur vacancies (Sv) were synthesized according to a previous report [1]. The Sv-ZIS was prepared by a hydrothermal method, 0.8 mmol $Zn(CH_3COO)_2 \cdot 2H_2O$, 1.6 mmol $InCl_3$, and 6.4 mmol TAA were dispersed in 15 mL deionized water and 15 mL ethanol with vigorous stirring for 30 min. Then, the obtained solution was transferred into a 50 ml Teflon-lined autoclave and heated at 180 °C for 24 h. The sample was washed by deionized water and ethanol for several times and then vacuum drying. The synthesis of pure $ZnIn_2S_4$ nanosheet was similar to that of Sv-ZIS except that 3.2 mmol TAA was added in the reaction.

Synthesis of Mo-Sv-ZIS

The synthesis process is the same as that of Sv-ZIS. Different contents (1.6 mg, 3.3 mg, 6.6 mg, 8 mg) of Na_2MoO_4 were added as molybdenum source during the synthesis of Sv-ZIS, and the final product was expressed as x% Mo-Sv-ZIS (X =0.8, 1.5, 3, 5), where x is the nominal mass percentage of Mo to Sv-ZIS. The Mo-ZIS was based on the synthetic ZIS with the addition of 3.3 mg of Na_2MoO_4. When the doping concentration is lower than 0.8%, the low doping amount has negligible effect on the photocatalytic performance. When the doping concentration is higher than 5%, the change of photocatalytic performance is also not obvious.

Characterizations

X-ray diffraction (XRD) patterns were examined by a Bruker D8 diffractometer using Cu Ka radiation. The morphologies and microstructures of the samples were characterized by a scanning electron microscope (SEM, JEOL, JSM-6700F, Japan) and a transmission electron microscope (TEM, FEI, JEM-2100PLUS, Japan). ICP-MS measurement was conducted on NexION 2000, PerkinElmer. Photoluminescence (PL) measurements were performed on a PL spectrometer

(FLS1000, Edinburgh). A 350 nm picosecond pulsed laser was used as the excitation source for the time-resolved PL measurements. X-ray photoelectron spectroscopy (XPS) was used to analyze the structure and composition of the samples by Thermo ESCALAB 250Xi with Al Ka source. The XPS spectra were calibrated through the standard C 1 s peak at 284.8 eV. Ultraviolet–visible diffuse reflectance (UV–vis) spectra in the range of 200-800 nm were recorded by a spectrophotometer (Cary5000, Varian, USA). Free radicals were explored by electron spin resonance spectra (ESR, JES-FA200, Japan).

Photoelectrochemical measurements

Electrochemical measurements were performed using an electrochemical analyzer (Autolab, PGSTAT 302 N) with a standard three-electrode system. 0.5 M Na_2SO_4 solution was used as the electrolyte for transient photocurrent and electrochemical impedance spectroscopy (EIS) measurements. The photocatalysts were coated on fluorine-doped tin oxide (FTO) glass with an area of 1 cm^2 in a three-electrode system. Platinum sheets and Ag/AgCl were used as counter and reference electrodes, respectively. Typically, 6 mg of catalyst was dispersed in 280 μL deionized water. And 20 μL Nafion solution was added after sonication to form a homogeneous suspension, which was then dropped evenly on the FTO glass and dried on a hot plate to ensure that the samples were snug on the FTO substrate. EIS was performed over a frequency range of 0.01 Hz to 100 kHz with an AC amplitude of 300 mV. Transient photocurrents were tested with illumination (300 W Xe lamp, 1.5 adaptive median (AM) filter) at 0.2 V bias.

Photocatalytic hydrogen production

Photocatalytic water splitting reaction over the photocatalysts was conducted in a pyrex top-irradiation reaction vessel connected to a closed glass gas circulation system with cooling water (6 °C) to maintain the reaction solution. Typically, 20 mg of photocatalyst powder was dispersed in 50 mL aqueous solution containing 10 vol% triethanolamine sacrificial agent. Before the test, the prepared suspension was evacuated for 30 min to completely remove the dissolved oxygen. A 300 W Xe lamp with a 420 nm cut-off filter was used as the visible light source. The amount of hydrogen evolution was analyzed by a gas chromatographer (GC7920, Beijing China Education Au-light Co. Ltd. China). The apparent quantum yield (AQY) for hydrogen evolution was measured under illumination of the simulated solar light with a band-pass filter (420 nm). Photon flux of the incident light was measured using an optical power meter (CEL-NP2000, Beijing

China Education Au-Light Co., Ltd). The AQY was calculated according to the following equation:

$$AQY(\%) = \frac{N_e}{N_p} = \frac{2 \times number\ of\ evolved\ H_2\ molecules}{number\ of\ incident\ photons} \times 100\%$$

$$= \frac{2 \times nH_2 \times N_A \times h \times c}{S \times P \times t \times \lambda} \times 100\%$$

where N_p is the total incident photons, N_e is the total reactive electrons, M is the amount of H_2 molecules, N_A is Avogadro constant, h is Planck's constant, c is the speed of light, S is the irradiation area, P is the intensity of irradiation light, t is the photoreaction time, and λ is the wavelength of the monochromatic light.

Calculation methods

The calculation adopted density functional theory (DFT) combined with mode conservation pseudopotential and plane wave method in CASTEP program. The conjugate gradient method was to minimize the total energy of the system to obtain a self-consistent ground state of the system. In the self-consistent field calculation, the electronic wave function of the system was obtained by the Pulay density mixing method. The Broyden, Fletcher, Goldfarb and Shannon (BFGS) methods were used to optimize the structure of the system. The generalized gradient approximation (GGA) and Perdew-Burke-Ernzerhof (PBE) functional combined. The plane wave cutoff energy was 500 eV. The Monkhorst Pack method was used to sample the Brillouin area, and the k-point grid was 5 × 5 × 1. In the process of structural optimization, the total energy convergence criterion of the system was 5.0×10^{-6} eV, the residual force convergence criterion was 0.01 eV×$Å^{-1}$, the atomic displacement convergence criterion was 5.0×10^{-4} Å, and the volume stress convergence criterion was 0.02 GPa. The cutoff energy of the plane wave and the value of the k-point grid ensured the convergence of the total energy of the system.

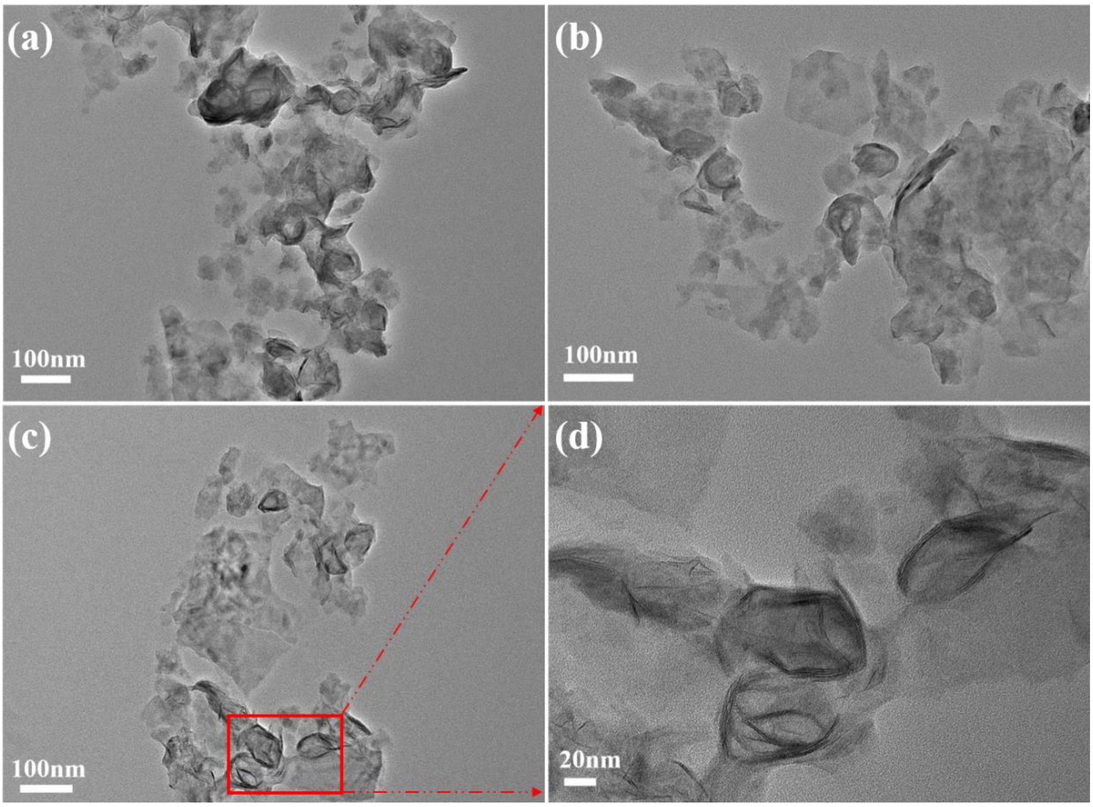

Figure. S1 (a-d) TEM images of 1.5% Mo-Sv-ZIS.

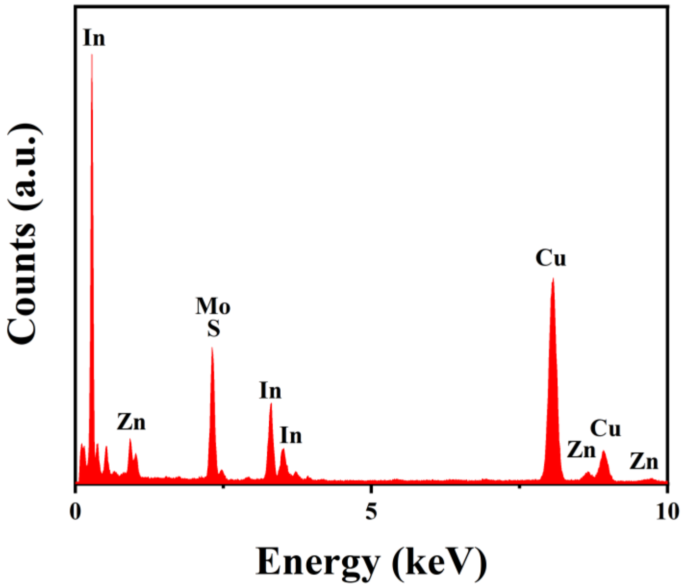

Figure. S2 EDS of the 1.5% Mo-Sv-ZIS shows the presence of Zn, In, S and Mo.

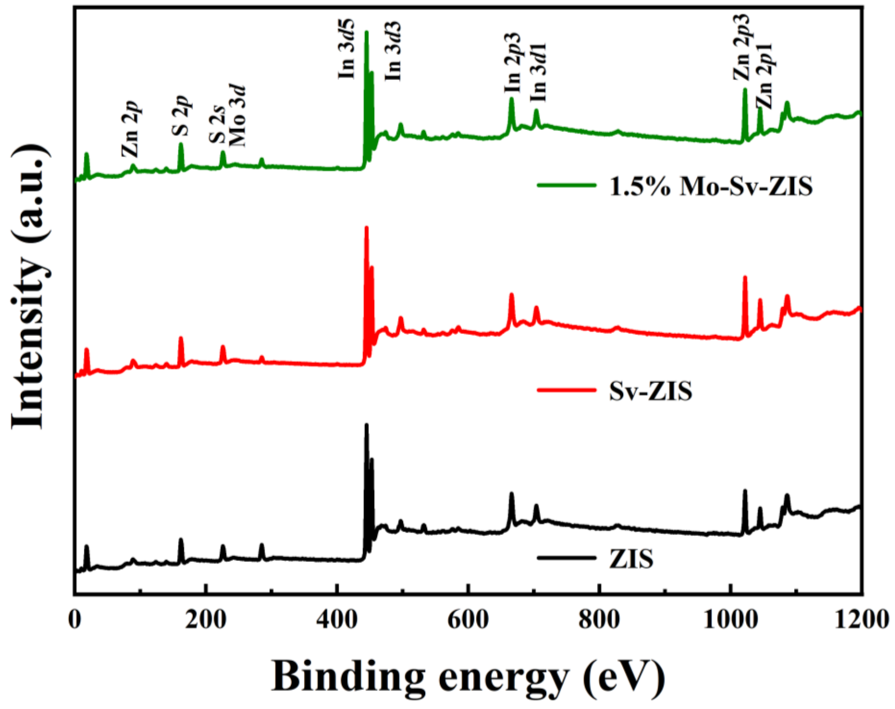

Figure. S3 Full survey XPS spectra of ZIS, Sv-ZIS and 1.5% Mo-Sv-ZIS.

Table. S1 Elemental compositions of ZIS, Sv-ZIS and 1.5%Mo-Sv-ZIS according to XPS.

**Theoretical chemical formula**	**ZIS**			**Sv-ZIS**			**1.5%Mo-Sv-ZIS**		
**Element**	Zn	In	S	Zn	In	S	Zn	In	S
**Atomic ratio**	17.67	28.41	53.97	20.57	25.81	53.62	20.38	26.48	53.15
**Actual chemical formula**	$Zn_{1.00}In_{1.60}S_{3.05}$			$Zn_{1.00}In_{1.25}S_{2.60}$			$Zn_{1.00}In_{1.30}S_{2.61}$		

Table. S2 Elemental contents of ZIS, Sv-ZIS and Mo-Sv-ZIS tested by ICP-MS.

**Sample**	**Zn(ppb)**	**In(ppb)**	**atomic ratio （Zn/In）**
**ZIS**	**21.90335**	**9.942138**	**3.838705**
**Sv-ZIS**	**14.6973**	**8.730693**	**2.933207**
**Mo-Sv-ZIS**	**9.910212**	**8.719684**	**1.980323**

As shown in Table S2, the Zn/In weight ratio of ZIS, Sv-ZIS and Mo-Sv-ZIS are 0.33, 0.22 and 0.15, respectively. Among them, the reduction of Zn and In content in Sv-ZIS compared to ZIS is mainly due to crystal defects (sulfur vacancies). The reduction of Zn content in Mo-Sv-ZIS compared to Sv-ZIS is mainly due to Mo atom substitution.

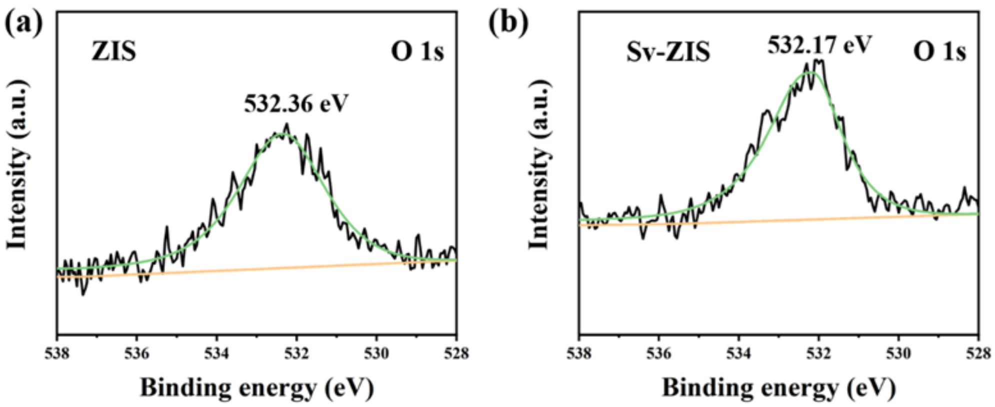

Figure. S4 High-resolution XPS spectra of O 1s of ZIS (a) and Sv-ZIS (b). The XPS O 1s peak of metal-O bonding typically locates at about 530 eV. The O 1s peak positions of ZIS and Sv-ZIS are 532.36 eV and 532.17 eV respectively. This proves that there is no metal-O bonding.

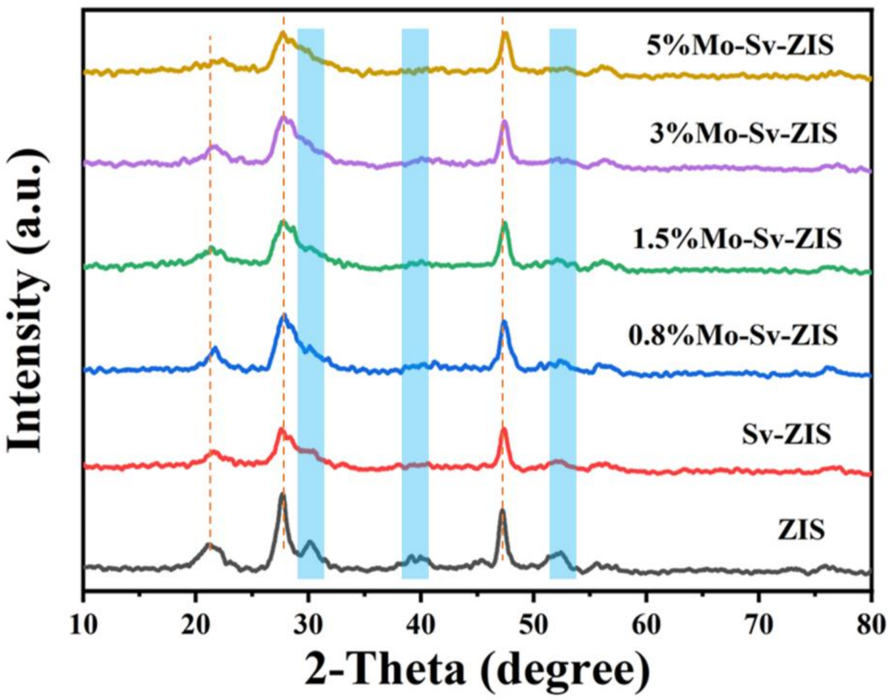

Figure. S5 XRD patterns of ZIS, Sv-ZIS and Mo-Sv-ZIS with different contents of Mo.

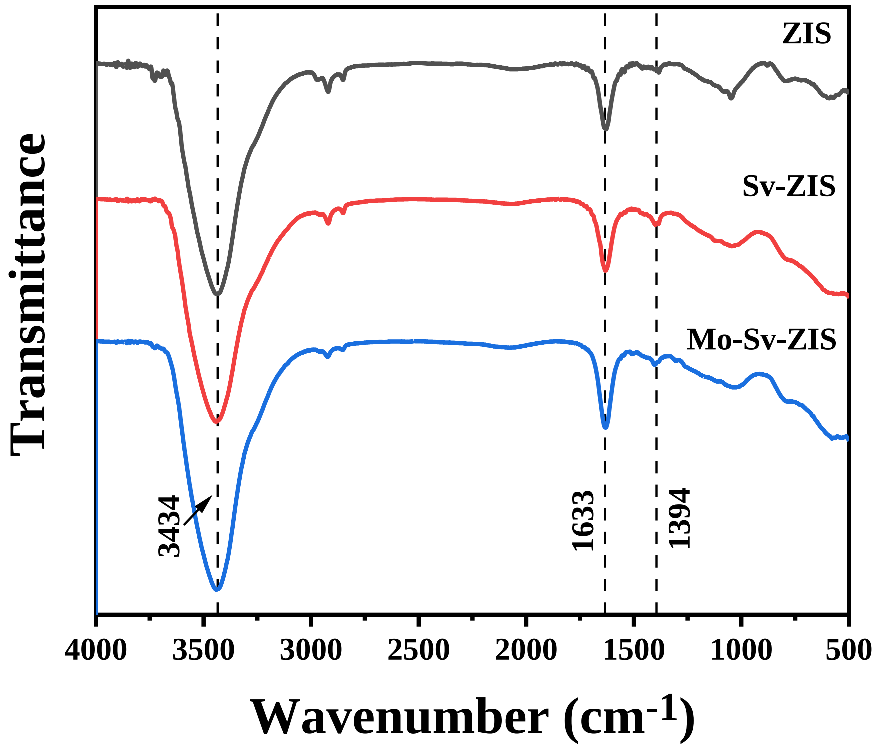

Figure. S6 FT-IR spectra of ZIS, Sv-ZIS and 1.5% Mo-Sv-ZIS.

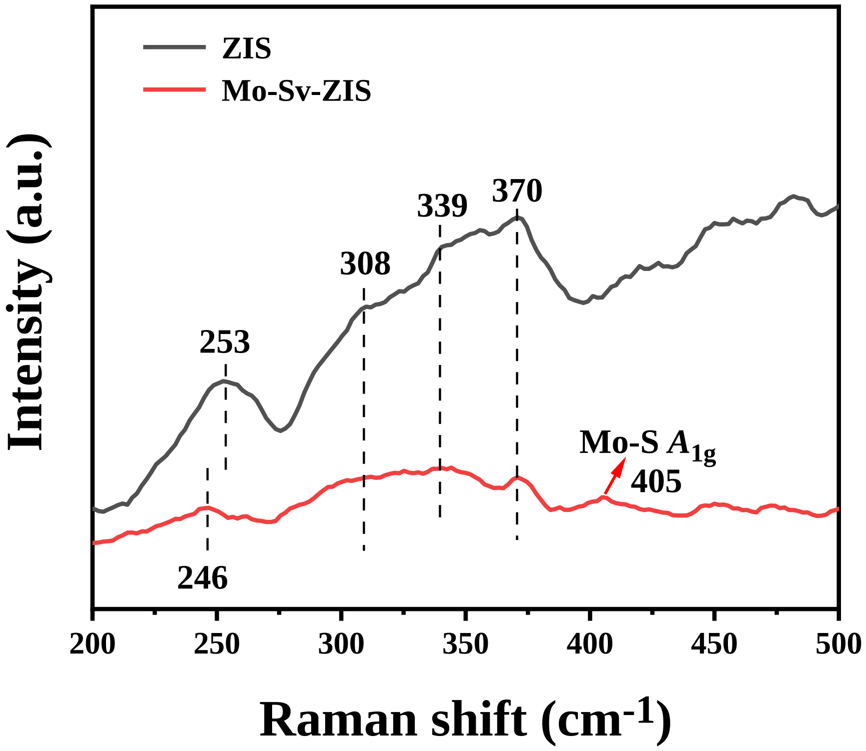

Figure S7. Raman spectra of ZIS and 1.5% Mo-Sv-ZIS.

Table S3 Exponential decay-fitting parameters for time resolved PL lifetime of ZIS, Sv-ZIS and 1.5% Mo-Sv-ZIS (465 nm).

	τ_1(ns)	A_1(%)	τ_2(ns)	A_2(%)	τ_3(ns)	A_3(%)	τ_A(ns)
**ZIS**	**0.254**	**36.6**	**2.747**	**58.2**	**12.483**	**5.2**	**5.331**
**Sv-ZIS**	**0.176**	**19.8**	**0.176**	**75.4**	**2.728**	**4.8**	**1.290**
**Mo-Sv-ZIS**	**0.082**	**99.9**	**0.199**	**0**	**2.847**	**0**	**0.082**

Table. S4 Exponential decay-fitting parameters for time resolved PL lifetime of ZIS, Sv-ZIS and 1.5% Mo-Sv-ZIS (528 nm).

	τ_1(ns)	A_1(%)	τ_2(ns)	A_2(%)	τ_3(ns)	A_3(%)	τ_A(ns)
**ZIS**	**0.360**	**65.6**	**3.843**	**20.8**	**3.841**	**13.6**	**3.315**
**Sv-ZIS**	**0.211**	**43.3**	**0.086**	**53.1**	**3.844**	**3.6**	**2.020**
**Mo-Sv-ZIS**	**0.059**	**94.5**	**0.242**	**5.2**	**4.397**	**0.3**	**0.856**

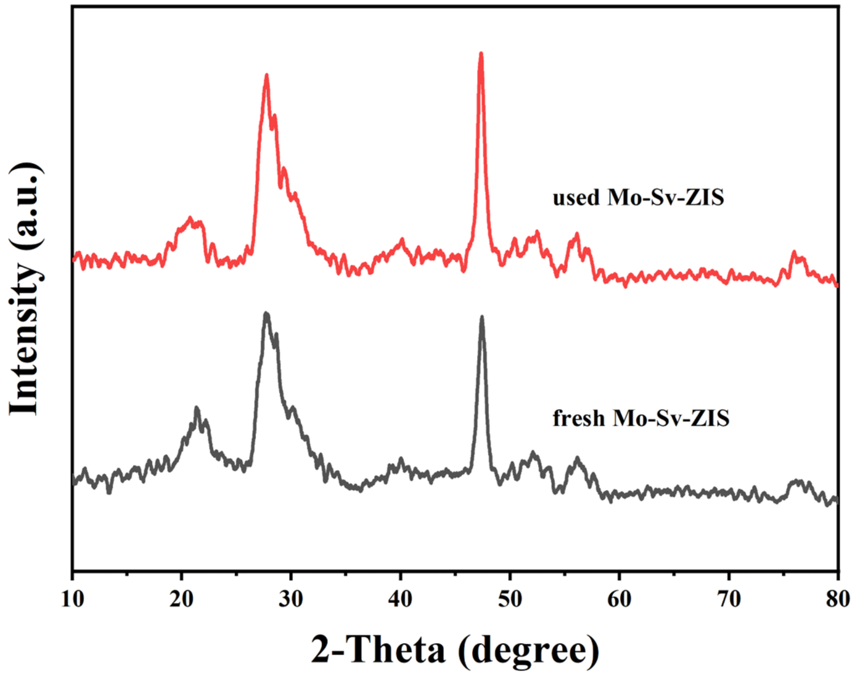

Figure. S8 XRD patterns of the as- prepared 1.5% Mo-Sv-ZIS and 1.5% Mo-Sv-ZIS after photocatalytic test.

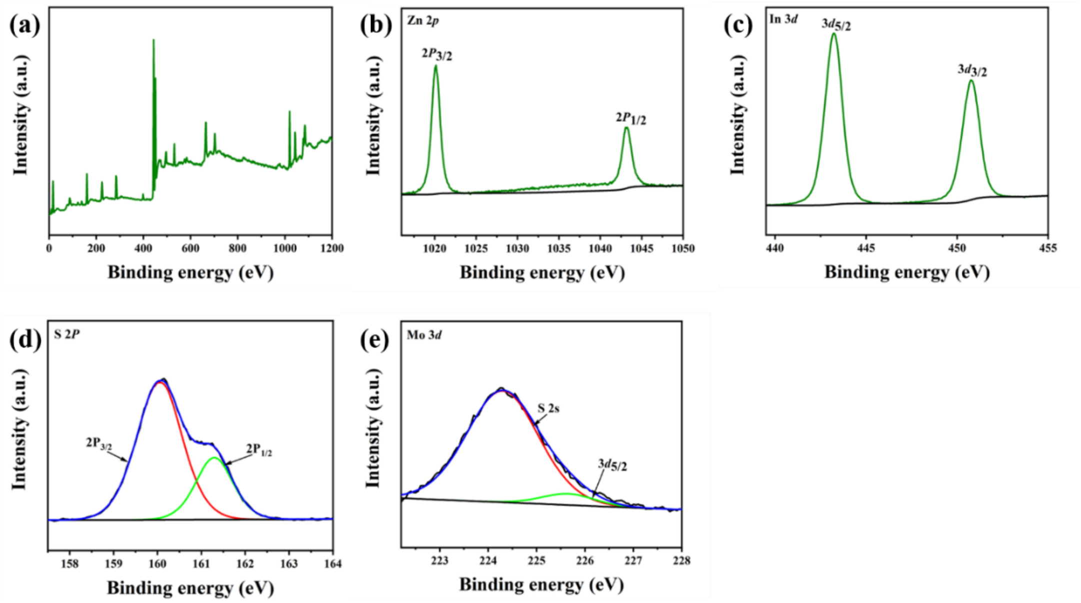

Figure. S9 XPS spectra of 1.5% Mo-Sv-ZIS after photocatalytic test. (a) Full survey XPS spectrum, (b) Zn 2*p*, (c) In 3*d*, (d) S 2*p* and (e) Mo 3*d*.

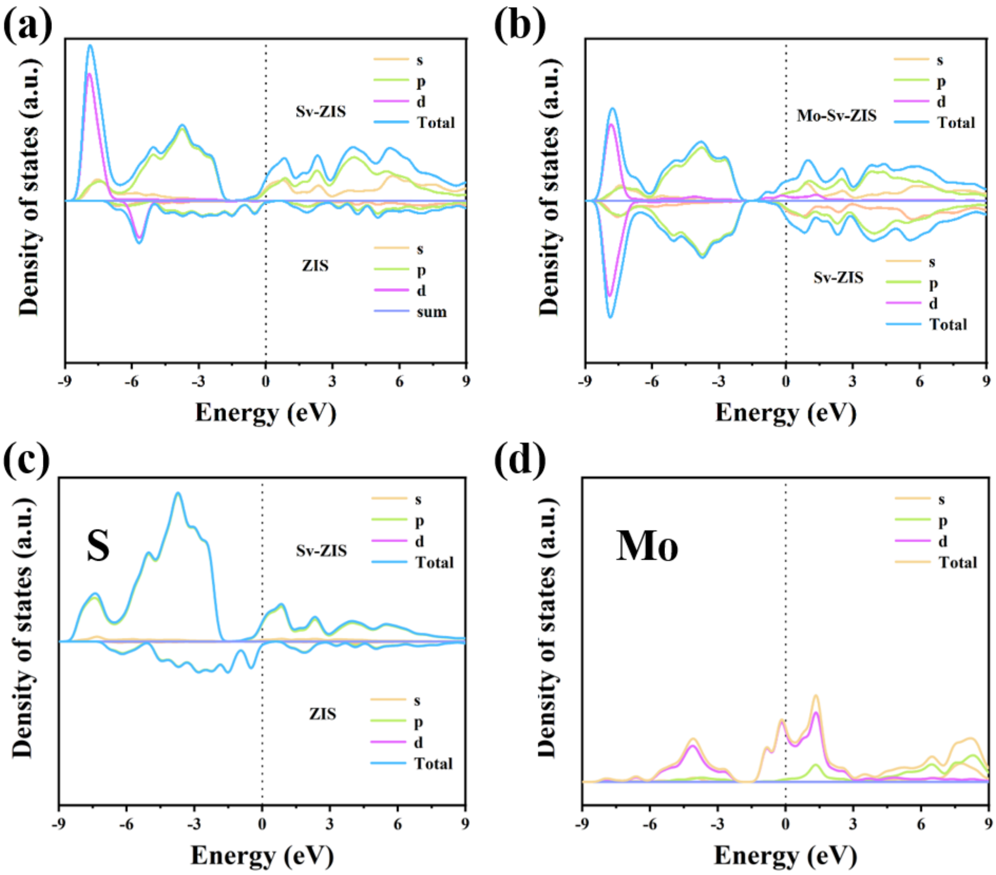

Figure. S10 The partial density of sates of (a) total orbit of ZIS and Sv-ZIS, (b) total orbit of Sv-ZIS and Mo-Sv-ZIS, (c) S atomic orbital of ZIS and Sv-ZIS, (d) Mo atomic orbital of Mo-Sv-ZIS.

As shown in Figure. S10 (a), the DOS of Sv-ZIS is higher than that of ZIS and the band gap is offset downward, which is consistent with the UPS and UV-vis results. The addition of S vacancies increases the density of states near the VBM of Sv-ZIS and causes a decrease in the CBM (Figure. S10 (c)). As shown in Figure. S10 (b), the DOS of Mo-Sv-ZIS at around the Fermi energy level increases significantly, which is mainly contributed by Mo atomic orbitals (Figure. S10(d)). The DOS of Mo atoms mainly concentrate in the CBM, which increases the electron accepting capacity of the CBM, causing the rise of CBM position. The calculation results are consistent with the band positions obtained by UPS and UV-vis.

Table S5. Impedance values of ZIS, Sv-ZIS and 1.5%Mo-Sv-ZIS obtained by fitting on the EIS results.

Sample	ZIS	Sv-ZIS	Mo-Sv-ZIS
Rs	4.27 Ω	4.12 Ω	3.97 Ω
Rp	111 Ω	81.8 Ω	58.8 Ω
CPE	0.00028 F	0.00023 F	0.00017 F

The EIS data were fitted through the equivalent circuit diagram shown in inset of Figure 6a. Among them, Rs represents the bulk solution resistance, and Rct corresponds to the charge-transfer resistance Rct.

AQY calculation process:

$$AQY(\%) = \frac{N_e}{N_p} = \frac{2 \times number\ of\ evolved\ H_2\ molecules}{number\ of\ incident\ photons} \times 100\%$$

$$= \frac{2 \times nH_2 \times N_A \times h \times c}{S \times P \times t \times \lambda} \times 100\%$$

When λ = 420 nm

$$\text{AQY}=\frac{2\times100.6\times10^{-6}\times6.022\times10^{23}\times6.626\times10^{-34}\times3\times10^{8}}{0.075\times3600\times420\times10^{-9}}\times100\%=21.24\%$$

Table. S6 Comparison of the 1.5% Mo-Sv-ZIS with other $ZnIn_2S_4$ related photocatalysts.

**Photocatalyst**	**Reaction condition**	**H_2 production (μmol h^{-1} g^{-1})**	**AQY (%)**	**Ref**
Mo-Sv-$ZnIn_2S_4$	TEOA<br>20 mg catalyst	5739	21.24<br>(420 nm)	This work
Mo-$ZnIn_2S_4$	TEOA<br>20 mg catalyst	4620	N/A	[2]
Ni-$ZnIn_2S_4$	TEOA<br>50 mg catalyst	5430	16.26<br>(420 nm)	[3]
Sv-$ZnIn_2S_4$<br>/CNTs/RP	Na_2S/Na_2SO_3<br>30 mg catalyst	1639.9	N/A	[4]
$NiTiO_3/ZnIn_2S_4$	TEOA<br>10 mg catalyst	4430	N/A	[5]
$FeWO_4/ZnIn_2S_4$	Na_2S/Na_2SO_3<br>50 mg catalyst	3531.2	19<br>(420 nm)	[6]
Pd@UiO-66-<br>NH_2@$ZnIn_2S_4$	TEOA<br>20 mg catalyst	5260	N/A	[7]
$ZnWO_4/ZnIn_2S_4$	methanol<br>20 mg catalyst	4925.3	N/A	[8]
Mo-$ZnIn_2S_4/MoO_3$	TEOA<br>10 mg catalyst	5500	4.82<br>(420 nm)	[9]
$Ta_3N_5/ZnIn_2S_4$	Na_2S/Na_2SO_3<br>30 mg catalyst	637.18	4.73<br>(420 nm)	[10]
BC/$ZnIn_2S_4$	LA<br>4 mg catalyst	4466	0.45<br>(420 nm)	[11]
$NiMoO_4$@Mo-<br>$ZnIn_2S_4$-Ni	TEOA<br>10 mg catalyst	5140	3.82<br>(420 nm)	[12]
Pd@TiO_2@$ZnIn_2S_4$	TEOA<br>20 mg catalyst	5350	N/A	[13]
Co-$ZnIn_2S_4$	TEOA<br>10 mg catalyst	2823	4.74<br>(420 nm)	[14]

SI References

1. Du, C.; Zhang, Q.; Lin, Z.; Yan, B.; Xia, C.; Yang, G. Half-unit-cell $ZnIn_2S_4$ monolayer with sulfur vacancies for photocatalytic hydrogen evolution. *Applied Catalysis B: Environmental* **2019**, *248*, 193-201, doi:10.1016/j.apcatb.2019.02.027.
2. Xing, F.; Liu, Q.; Huang, C. Mo-Doped $ZnIn_2S_4$ Flower-Like Hollow Microspheres for Improved Visible Light - Driven Hydrogen Evolution. *Solar RRL* **2019**, *4*, 1900483, doi:10.1002/solr.201900483.
3. Qiu, B.; Huang, P.; Lian, C.; Ma, Y.; Xing, M.; Liu, H.; Zhang, J. Realization of all-in-one hydrogen-evolving photocatalysts via selective atomic substitution. *Applied Catalysis B: Environmental* **2021**, *298*, 120518, doi:10.1016/j.apcatb.2021.120518.
4. Liu, L.; Liu, J.; Yang, W.; Wan, J.; Fu, F.; Wang, D. Constructing a Z-scheme $ZnIn_2S_4$-S/CNTs/RP nanocomposite with modulated energy band alignment for enhanced photocatalytic hydrogen evolution. *J Colloid Interface Sci* **2022**, *608*, 482-492, doi:10.1016/j.jcis.2021.09.145.
5. Dhingra, S.; Sharma, M.; Krishnan, V.; Nagaraja, C.M. Design of noble metal-free $NiTiO_3$/ $ZnIn_2S_4$ heterojunction photocatalyst for efficient visible-light-assisted production of H_2 and selective synthesis of 2,5-Bis(hydroxymethyl)furan. *J Colloid Interface Sci* **2022**, *615*, 346-356, doi:10.1016/j.jcis.2022.01.190.
6. Kong, D.; Hu, X.; Geng, J.; Zhao, Y.; Fan, D.; Lu, Y.; Geng, W.; Zhang, D.; Liu, J.; Li, H.; et al. Growing $ZnIn_2S_4$ nanosheets on $FeWO_4$ flowers with p-n heterojunction structure for efficient photocatalytic H_2 production. *Applied Surface Science* **2022**, *591*, 153256, doi:10.1016/j.apsusc.2022.153256.
7. Cao, M.; Yang, F.; Zhang, Q.; Zhang, J.; Zhang, L.; Li, L.; Wang, X.; Dai, W.-L. Facile construction of highly efficient MOF-based Pd@UiO-66-NH_2@ $ZnIn_2S_4$ flower-like nanocomposites for visible-light-driven photocatalytic hydrogen production. *Journal of Materials Science & Technology* **2021**, *76*, 189-199, doi:10.1016/j.jmst.2020.11.028.
8. Dai, M.; He, Z.; Zhang, P.; Li, X.; Wang, S. $ZnWO_4$- $ZnIn_2S_4$ S-scheme heterojunction for enhanced photocatalytic H_2 evolution. *Journal of Materials Science & Technology* **2022**, *122*, 231-242, doi:10.1016/j.jmst.2022.02.014.
9. Su, H.; Lou, H.; Zhao, Z.; Zhou, L.; Pang, Y.; Xie, H.; Rao, C.; Yang, D.; Qiu, X. In-situ Mo doped $ZnIn_2S_4$ wrapped MoO_3 S-scheme heterojunction via Mo-S bonds to enhance photocatalytic HER. *Chemical Engineering Journal* **2022**, *430*, 132770, doi:10.1016/j.cej.2021.132770.
10. Zhan, X.; Zheng, Y.; Li, B.; Fang, Z.; Yang, H.; Zhang, H.; Xu, L.; Shao, G.; Hou, H.; Yang, W. Rationally designed Ta_3N_5/ $ZnIn_2S_4$ 1D/2D heterojunctions for boosting Visible-Light-driven hydrogen evolution. *Chemical Engineering Journal* **2022**, *431*, 134053, doi:10.1016/j.cej.2021.134053.
11. Bhavani, P.; Praveen Kumar, D.; Hussain, M.; Aminabhavi, T.M.; Park, Y.-K. Eco-friendly rice husk derived biochar as a highly efficient noble Metal-Free cocatalyst for high production of H_2 using solar light irradiation. *Chemical Engineering Journal* **2022**, *434*, 134743, doi:10.1016/j.cej.2022.134743.
12. Su, H.; Rao, C.; Zhou, L.; Pang, Y.; Lou, H.; Yang, D.; Qiu, X. Mo-Doped/Ni-supported $ZnIn_2S_4$-wrapped $NiMoO_4$ S-scheme heterojunction photocatalytic reforming of lignin into hydrogen. *Green Chemistry* **2022**, *24*, 2027-2035, doi:10.1039/d1gc04397h.

13. She, P.; Qin, J.S.; Sheng, J.; Qi, Y.; Rui, H.; Zhang, W.; Ge, X.; Lu, G.; Song, X.; Rao, H. Dual-Functional Photocatalysis for Cooperative Hydrogen Evolution and Benzylamine Oxidation Coupling over Sandwiched-Like Pd@TiO_2 @ $ZnIn_2S_4$ Nanobox. *Small* **2022**, *18*, e2105114, doi:10.1002/smll.202105114.
14. Xue, J.; Liu, H.; Zeng, S.; Feng, Y.; Zhang, Y.; Zhu, Y.; Cheng, M.; Zhang, H.; Shi, L.; Zhang, G. Bifunctional Cobalt-Doped $ZnIn_2S_4$ Hierarchical Nanotubes Endow Noble-Metal Cocatalyst-Free Photocatalytic H_2 Production Coupled with Benzyl Alcohol Oxidation. *Solar RRL* **2022**, *6*, 2101042, doi:10.1002/solr.202101042.
